# New diabatic potential energy surfaces of the NaH_2_ system and dynamics studies for the Na(3p) + H_2_ → NaH + H reaction

**DOI:** 10.1038/s41598-018-35987-z

**Published:** 2018-12-19

**Authors:** Shufen Wang, Zijiang Yang, Jiuchuang Yuan, Maodu Chen

**Affiliations:** 1 Key Laboratory of Materials Modification by Laser, Electron, and Ion Beams (Ministry of Education), School of Physics, Dalian University of Technology, Dalian, 116024 P.R. China; 2 State Key Laboratory of Molecular Reaction Dynamics, Dalian Institute of Chemical Physics, Chinese Academy of Science, Dalian, 116023 P.R. China

## Abstract

The Na(3p) + H_2_(X^1^Σ_g_^+^) → NaH(X^1^Σ^+^) + H(^2^S) reaction plays an important role in the field of diabatic reaction dynamics. A set of new diabatic potential energy surfaces (PESs) of the NaH_2_ system are structured, which include the diabatic coupling between the lowest two adiabatic states. The electronic structure calculations are performed on the multi-reference configuration interaction level with the cc-pwCVQZ and aug-cc-PVQZ basis sets for Na and H atoms. 32402 geometries are chosen to construct the diabatic data by a unitary transformation based on the molecular property method. The diabatic matrix elements of ${{\boldsymbol{V}}}_{{\bf{11}}}^{{\boldsymbol{d}}}$, ${{\boldsymbol{V}}}_{{\bf{22}}}^{{\boldsymbol{d}}}$ and ${{\boldsymbol{V}}}_{{\bf{12}}}^{{\boldsymbol{d}}}$ (${{\boldsymbol{V}}}_{{\bf{21}}}^{{\boldsymbol{d}}}$) are fitted by the artificial neural network model. The spectroscopic constants of diatoms obtained from the present PESs are consistent with the experimental data. The topographical and intersection characteristics of the ${{\boldsymbol{V}}}_{{\bf{11}}}^{{\boldsymbol{d}}}$ and ${{\boldsymbol{V}}}_{{\bf{22}}}^{{\boldsymbol{d}}}$ surfaces are discussed. Based on the new PESs, the time-dependent quantum wave packet calculations are carried out to study the reaction mechanism of the title reaction in detail.

## Introduction

The interactions between electronically excited alkali atoms and hydrogen molecule, including both no-reactive quenching and chemical reactions, have become an interesting topic due to the special advantage for understanding diabatic processes. For the collisions of excited alkali atoms with H_2_, the diabatic couplings necessarily occur, and the conical intersections arise to connect two or more surfaces. Among the issues of reactive collision for these alkali elements, the reactions of K^1–4^, Rb^5,6^, and Cs^7–9^ with H_2_ proceed by a harpoon model, whereas the reactions of Li^10–13^ and Na^14–16^ with H_2_ follow an insertion mechanism.

The NaH_2_ presents a simple prototype of such collision systems, and numerous experimental results^15–22^ about the collisions between excited sodium atom and hydrogen molecule are available. Botschwina *et al*.^17^ performed crossed molecular beam experiments on the Na(3p) + H_2_ → Na(3 s) + H_2_ process. The results of quenching cross section and vibrational energy distribution were presented in their work. Bilililign *et al*.^15^ studied the Na(4p) + H_2_ → NaH + H photochemistry reaction by using the “half-collision” pump-probe technique. They observed a bimodal rotational distribution of the NaH product: the minor peak at lower rotational states is due to the repulsive interaction, while the major peak at higher rotational states is attributed to the attractive interaction. Motzkus *et al*.^20^ applied three different nonlinear optical techniques, including coherent anti-Stokes Raman scattering (CARS), resonance-enhanced CARS, and degenerate four-wave mixing, to compare the Na(4p) + H_2_ and Na(3p) + H_2_ reaction processes. This experiment showed the formation of NaH via the Na(3p) + H_2_ reaction follows a two-step mechanism, which is opposite to the direct reaction of Na(4p) + H_2._ In 2008, Chang and co-workers^22^ studied the rotational and vibrational state distributions of NaH in the reactions of high exited states Na(4^2^S, 3^2^D and 6^2^S) with H_2_ by using the pump-probe technique. The authors concluded that the Na(6^2^S) reaction has a dramatically reduced ionization energy, and the corresponding reaction pathway maybe prefer a harpoon model via a near collinear configuration. For the Na(6^2^S) + H_2_ reaction, the valence electron of Na hopping mechanism is involved to form an ion-pair Na^+^H_2_^−^ intermediate.

Extensive theoretical studies^23–27^ have also been performed on the NaH_2_ system based on several potential energy surfaces (PESs)^17,28–33^, which were concentrated on studying the effect of conical intersection and the processes of electronic-to-rovibrational energy transfer. In 1982, Donald *et al*.^28^ calculated the lowest three PESs and diabatic coupling for the Na(3p) + H_2_ quenching process by using the diatomics-in-molecules (DIM) method. Then they used a new parametrization for the diabatic potential energy curves of NaH^29^ to optimize the preceding PESs, which are only suitable for studying the non-reactive quenching of the Na(3p) + H_2._ In 1999, Michael *et al*.^30^ structured an analytical potential energy matrix of the NaH_2_ system for the lowest two states based on MP2 calculations, which only include a small number of *ab initio* energy points near the region of conical intersection. The potential energy matrix can be applied in reaction dynamics calculations of the diabatic processes.

The reaction of lowest electronically excited state Na(3p) with H_2_ plays an important role for studying diabatic reaction dynamics correlated with two adiabatic states. The reaction starts at the adiabatic 2^2^A′ surface and then intersects the adiabatic 1^2^A′ surface to enter into the product channel. The formation of NaH molecule by this reaction follows a two-steps process. The first step is the quenching between Na(3p) and H_2_,

$${\rm{Na}}(3{\rm{p}})+{{\rm{H}}}_{2}(v^{\prime} =0)\to {\rm{Na}}(3{\rm{s}})+{{\rm{H}}}_{2}(v^{\prime} \ge 1),$$

and then Na(3p) collides with the vibrationally excited H_2_,

$${\rm{Na}}(3{\rm{p}})+{{\rm{H}}}_{2}(v^{\prime} \ge 1)\to {\rm{NaH}}+{\rm{H}}.$$

The correctness of this process has been proved by rate equation model^21^. However, the quantum dynamics study of the Na(3p) + H_2_(X^1^Σ_g_^+^) → NaH(X^1^Σ^+^) + H(^2^S) reaction has not been reported. For the title reaction, the early diabatic PESs may be not accurate enough to investigate the state-to-state reaction dynamics due to the limit of computational conditions. Fortunately, the recent advances in *ab initio* theory and the neural network (NN) model make it possible to obtain accurate global PESs. In this work, a set of new diabatic PESs involved two lowest adiabatic states (1^2^A′ and 2^2^A′) and the coupling between them are structured with the NN method. To guarantee the accuracy of the new diabatic PESs, numerous high precision single point energies in a wide coordinate range are calculated, which are used to construct the energy matrix in the diabatic representation by the molecular property method. Then the time-dependent wave packet (TDWP) calculations are carried out on the new diabatic PESs to obtain the quantum dynamics information of the title reaction.

## Results

### Topographical features of the PESs

**Figure 1:**
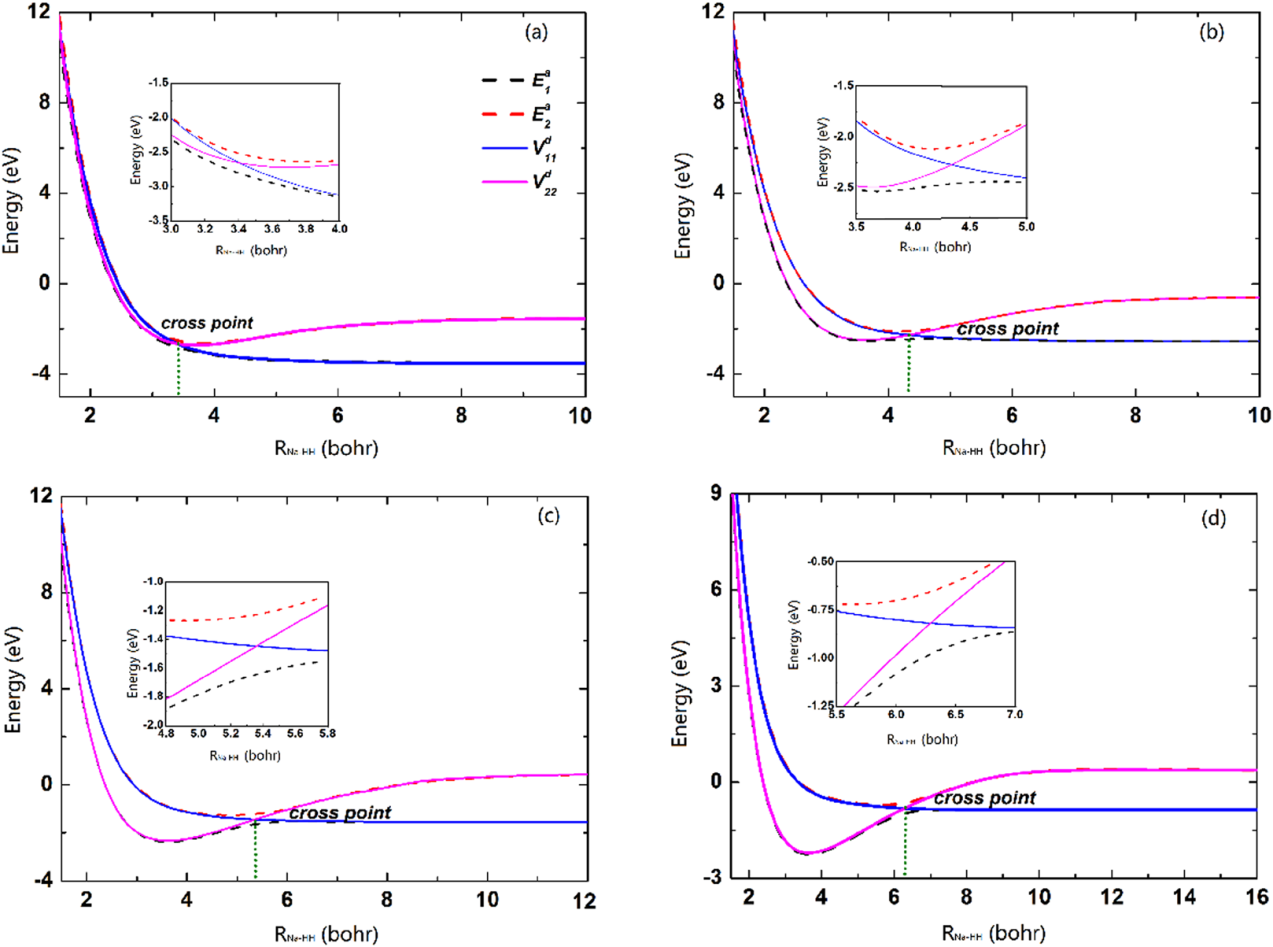
Potential energy curves in the diabatic and adiabatic representations as a function of *R*_Na-HH_ for (**a**) *r*_HH_ = 2.1 *a*_0_, (**b**) 2.5 *a*_0_, (**c**) 3.0 *a*_0_ and (**d**) 3.5 *a*_0_ in *C*_*2v*_ geometry.

Figure 1 presents potential energy curves in the adiabatic and diabatic representations as a function of *R*_Na-HH_ at four fixed internuclear distances of HH (*r*_HH_ = 2.1, 2.5, 3.0, 3.5 bohr) in *C*_*2v*_ geometry. The electronic symmetry of the ground and first excited states turn into 1^2^A_1_ and 1^2^B_2_ in *C*_2v_ geometry, respectively. It can be seen that the adiabatic potentials strongly avoid, while the curves in the diabatic representation cross over each other smoothly near the crossing point. The crossing point located at larger *R*_Na-HH_ with the *r*_HH_ increasing. Moreover, the adiabatic and daibatic potential energy curves are overlapping when *R*_Na-HH_ is far away from the crossing point, implying the electronic coupling is zero in the asymptotic region.

**Figure 2:**
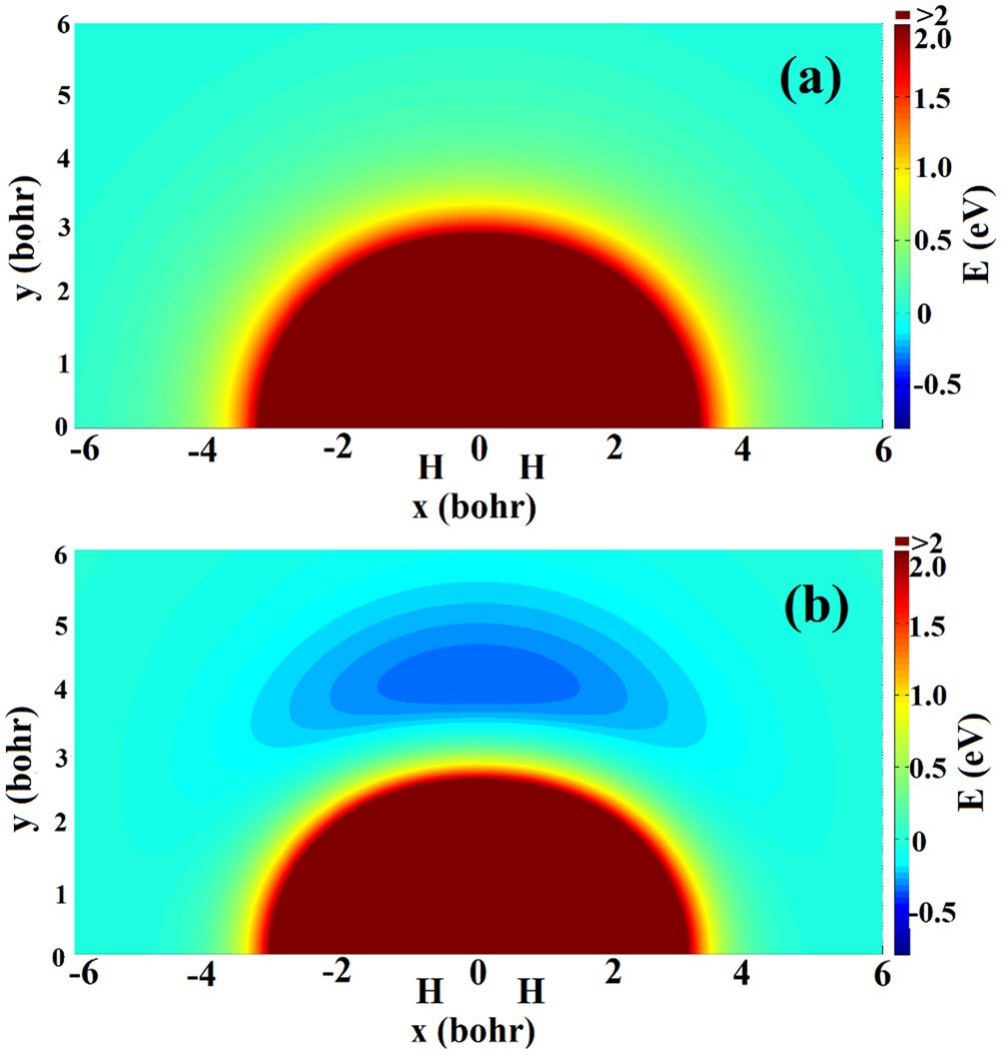
Contour plots of the diabatic surfaces (**a**) ${V}_{11}^{d}$ and (**b**) ${V}_{22}^{d}$ for a Na atom moving around the H_2_ diatom fixed at the equilibrium distance.

**Figure 3:**
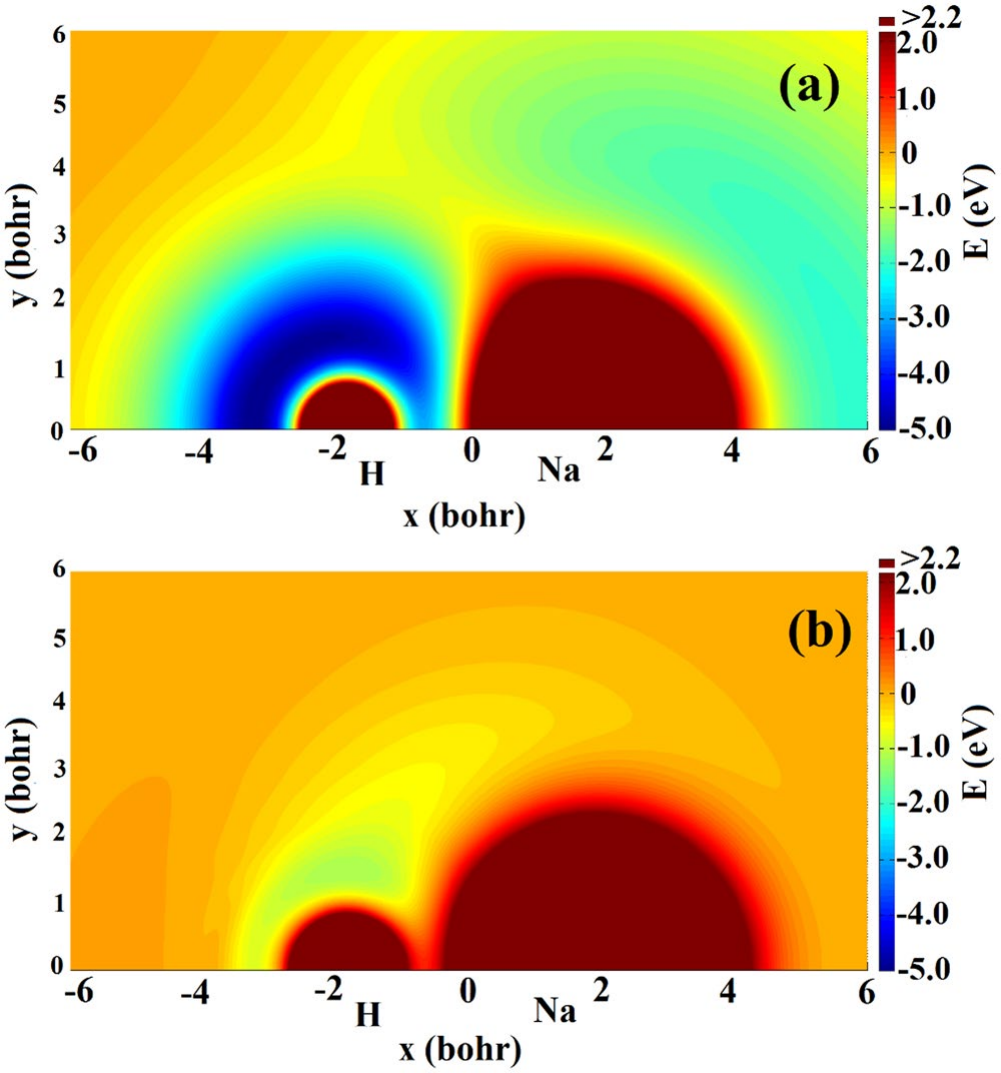
Contour plots of the diabatic surfaces (**a**) ${V}_{11}^{d}$ and (**b**) ${V}_{22}^{d}$ for a H atom moving around the NaH diatom fixed at the equilibrium distance.

Figure 2(a,b) show equipotential energy contours of the ${V}_{11}^{d}$ and ${V}_{22}^{d}$ for a Na atom moving around the H_2_ molecule, respectively. The HH bond length is set to the equilibrium distance (1.401 bohr) corresponded the ground electronic state. No obvious well or barrier can be found on the ${V}_{11}^{d}$ surface, and the Na atom receives the repulsive interaction of the H_2_ molecule. There exists a wide well around the H_2_ molecule on the ${V}_{22}^{d}$ surface, and the deepest structure is about 0.36 eV located at x = 0.0 *a*_0_, y = 4.1 *a*_0_. The Na atom can be attracted by the well to form the metastable intermediate when it moves to the H_2_ molecule, and the intermediate enters product channel to dissociate to the NaH + H on the ${V}_{22}^{d}$ surface. Similar contours to Fig. 2 but for a H atom moving around the NaH molecule are displayed in Fig. 3(a,b). The NaH bond length is set to its ground electronic state equilibrium distance (3.639 bohr). For the ${V}_{11}^{d}$ surface, there are two wells, which close to Na atom and H atom, respectively. The depth of the well around H atom reaches 4.97 eV, indicating the single H atom is more easily attracted on the side of H atom on the ${V}_{11}^{d}$. For the ${V}_{22}^{d}$ surface, there is a 1.16 eV deep well around H atom. The single H atom feels the repulsive force of NaH molecule when it is near Na atom, and the well will attract the single H when it is on the side of H atom.

**Figure 4:**
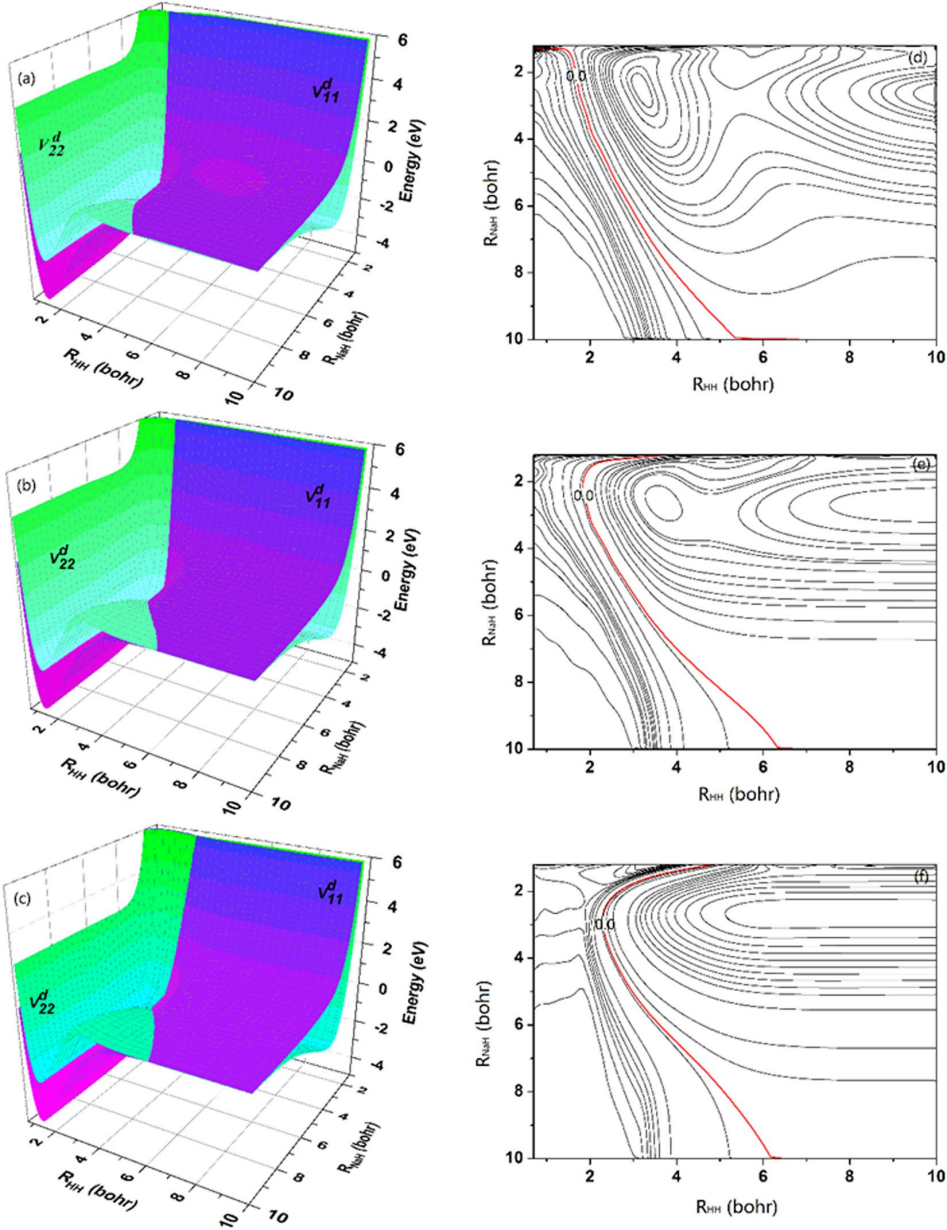
Three dimension diabatic PESs of ${V}_{11}^{d}$ and ${V}_{22}^{d}$ for the fixed Na-H-H angle of (**a**) 60°, (**b**) 90° and (**c**) 180°. The corresponding contour plots of $({V}_{22}^{d}-{V}_{11}^{d})$ are presented in (**d**), (**e**) and (**f**), and the intersection of two diabatic surfaces is described by red line.

**Figure 5:**
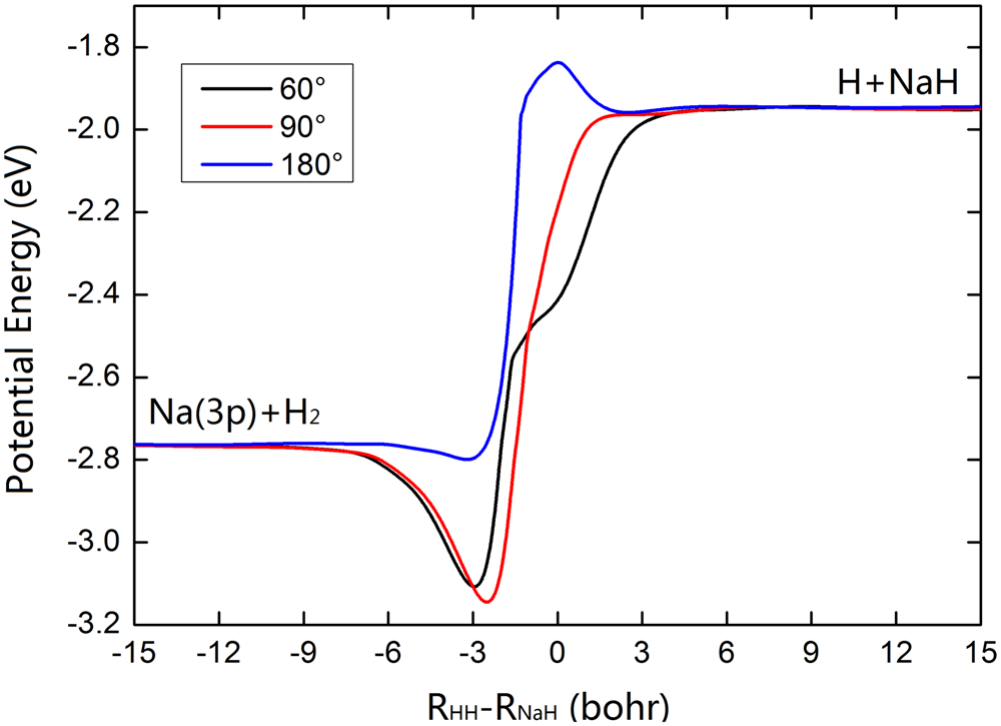
Minimum energy paths of the Na(3p) + H_2_(X^1^Σ_g_^+^) → NaH(X^1^Σ^+^) + H(^2^S) reaction for the fixed Na-H-H angle of 60°, 90° and 180°.

The diabatic ${V}_{11}^{d}$ and ${V}_{22}^{d}$ surfaces and the contour maps of $({V}_{22}^{d}-{V}_{11}^{d})$ at three Na-H-H angles (60°, 90° and 180°) are presented in Fig. 4. The three-dimensional plots show the fitted PESs are smooth over the whole coordinate space. There is a valley corresponding to Na (3 s) + H_2_ channel on the ${V}_{11}^{d}$_._ For the ${V}_{22}^{d}$ surface, two valleys can be found on the left and right, which represent the Na(3p) + H_2_ and NaH + H channels, respectively. Thus in the diabatic representation, the ${V}_{22}^{d}$ surface provides a direct path from Na(3p) + H_2_ to NaH + H. For the lower panel of Fig. 4, the red line depicts the position of intersection between the ${V}_{11}^{d}$ and ${V}_{22}^{d}$ PESs. The difference between two diabatic PESs increases in the region away from the intersection position, and this feature is consistent with the results of Fig. 1. The minimum reaction paths for the title reaction at three Na-H-H angles (60°, 90° and 180°) on the ${V}_{22}^{d}$ PES are presented in Fig. 5. For the Na-H-H angle of 60° and 90°, there exist a well along the reaction path, which corresponds the complex-forming reaction. For the Na-H-H angle of 180°, there exists a 0.1 eV height barrier, which implies it is very difficult to initiate the title reaction through the Na-H-H collinear path. Namely, the reaction of Na(3p) with H_2_ is dominated by an insertion approach. Combined with previous studies, it could conclude that the reactions of low-lying electronic states Na with H_2_ follow an insertion model, whereas the reactions of highly-excited states Na with H_2_ could favor the harpoon-type mechanism. Moreover, taking into account the zero point energies of H_2_ (0.271 eV) and NaH (0.072 eV) molecules, the endothermicity for forming the NaH molecule is about 0.63 eV.

### Dynamical calculations

**Figure 6:**
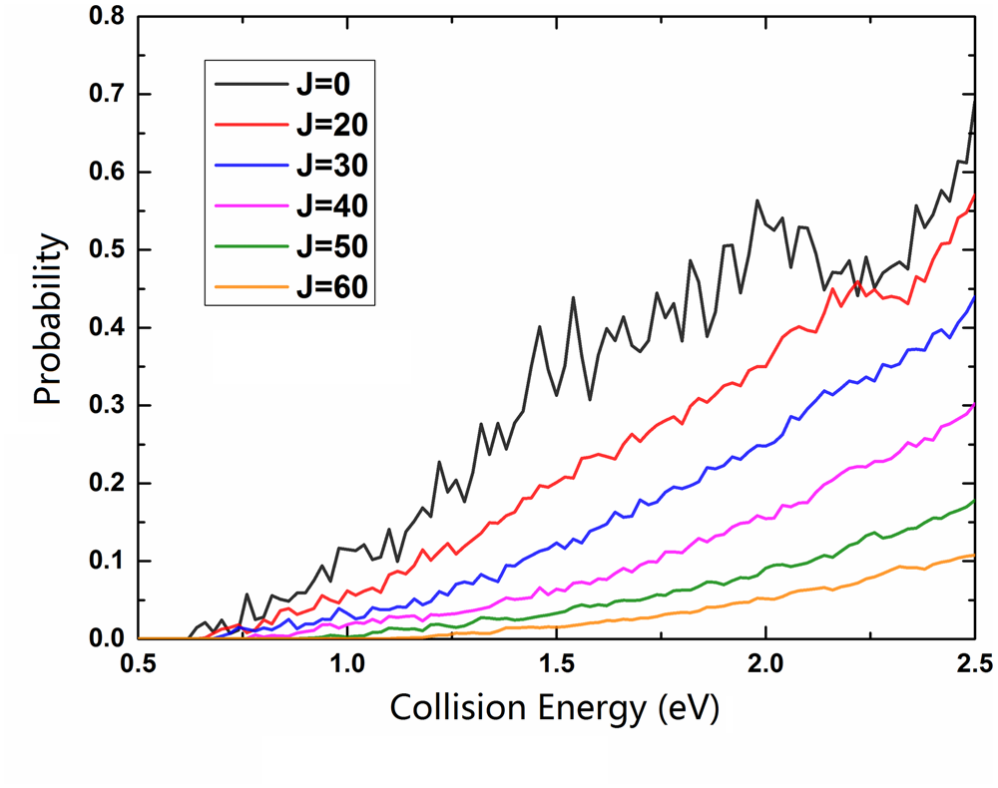
Total reaction probabilities of the Na(3p) + H_2_(X^1^Σ_g_^+^) → NaH(X^1^Σ^+^) + H(^2^S) reaction at *J* = 0, 20, 30, 40, 50 and 60.

Figure 6 describes the collision energy dependence of total reaction probabilities for the title reaction at six different *J* values. The curve of *J* = 0 shows the reaction threshold is about 0.63 eV, corresponded with the endothermicity calculated on the PESs, implying the title reaction occurs by a barrierless path in the product channel. The threshold increases at large *J* value due to the increasing centrifugal barrier. Some sharp resonance peaks can be found due to the potential well on ${V}_{22}^{d}$ PES, which gives rise to the formation of intermediate complex. Moreover, the reaction probabilities decrease and the resonance structures become less pronounced as *J* value increasing, which is attributed to the large centrifugation potential leads to more Na atoms entrance the product channel without the well, thus the lifetime of complex becomes shorter.

**Figure 7:**
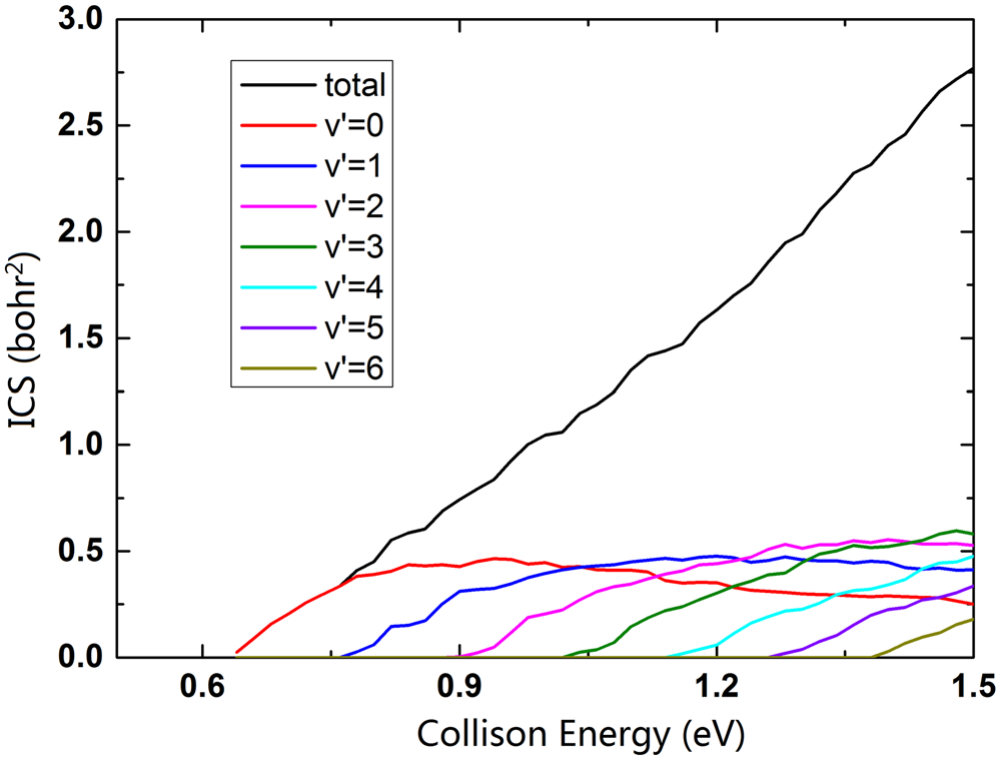
Total and vibrational states resolved ICSs of the Na(3p) + H_2_(X^1^Σ_g_^+^) → NaH(X^1^Σ^+^) + H(^2^S) reaction.

In the TDWP calculations, the maximum *J* value is calculated up to 70, which can ensure the convergence of integral cross sections (ICSs) and differential cross sections (DCSs) when the collision energy is below 1.5 eV. The ICS results of the Na(3p) + H_2_ reaction are similar to the Li + H_2_ reaction^34^. The ICSs curves are relatively smooth, compared with the oscillating reaction probability curves. The total ICS and several vibrational states (*v*′ = 0 − 6) ICSs of the title reaction are displayed in Fig. 7. The total ICS value steeply rises at the selected energy region, and the six vibrational excitation channels of the NaH molecule are opened successively. The ICSs of vibrational excitation states keep growth at the collision energy below 1.5 eV. The ground vibrational state ICS rises up to the collision energy reaches 0.94 eV, and then decreases gradually at the energy region from 0.94 to 1.5 eV. It implies more energy are transformed into the internal energy of the NaH molecule, and the product are excited to higher vibrational states.

**Figure 8:**
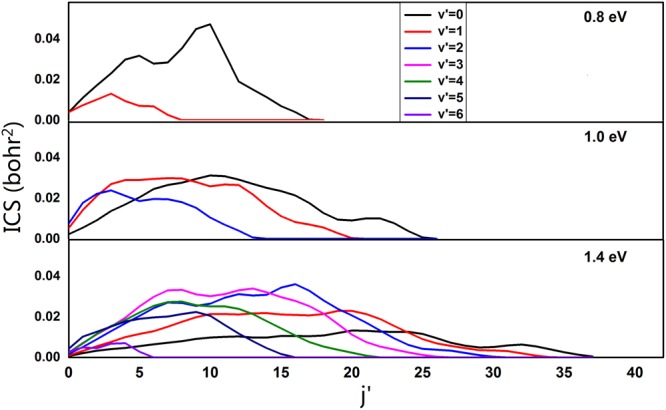
Rovibrational resolved ICSs of the Na(3p) + H_2_(X^1^Σ_g_^+^) → NaH(X^1^Σ^+^) + H(^2^S) reaction at three collision energies (0.8 eV, 1.0 eV and 1.4 eV).

Furthermore, the product rotational distribution for three selected collision energies (0.8, 1.0, 1.4 eV) are calculated, which are shown in Fig. 8. It can be seen that the rotational states of the product NaH molecule are inverted in all vibrational levels, and the range of rotational quantum number of the product NaH molecule becomes broader with increase of the collision energy. Since more energies can be transferred into the internal energy of the NaH molecule. In all cases, as vibrational quantum increases, the peak of rotational states distribution shifts to lower rotational quantum number. This is because the total energy is constant, and the internal energy shifts from rotation to vibration with increasing vibrational level.

**Figure 9:**
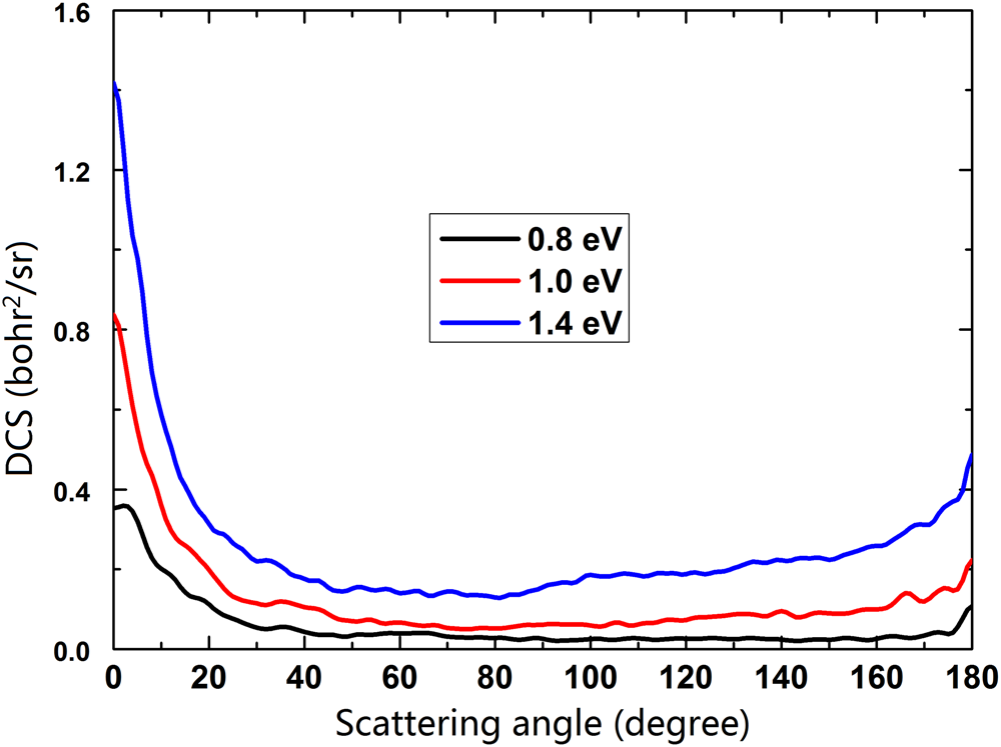
DCSs of the Na(3p) + H_2_(X^1^Σ_g_^+^) → NaH(X^1^Σ^+^) + H(^2^S) reaction at three collision energies (0.8 eV, 1.0 eV and 1.4 eV).

The DCS gives the product angular distribution of a reaction. Figure 9 shows the DCSs of the title reaction at three collision energies (0.8, 1.0, 1.4 eV). It is clear that the product NaH molecule tends to be forward scattered, which implies that the product NaH molecule prefers moving toward the initial direction of the reactant Na atom. This forward bias means that the reaction is dominated by the formation of short-lived complex. With the collision energy increasing, the forward scattered becomes more obvious due to the proportion of direct reactive mechanism increases at a high collision energy.

## Discussion

In the present work, a set of new global PESs for the NaH_2_ system are constructed in the diabatic representation, which are correlated with the adiabatic 1 A′state and 2 A′state. The *ab initio* calculations are performed at the internally contracted multi-reference configuration interaction level with the Davidson correction (icMRCI + Q). The aug-cc-PVQZ and cc-pWCVQZ and basis sets are adopted for H atom and Na atom, respectively. The diabatic matrix elements are generated by the transformation of *ab initio* data based on the molecular property method. A mass of geometries (32402) in a large coordinate space are selected to fit the diabatic PESs using the NN model, and the root mean squared errors (RMSEs) for the diabatic terms ${V}_{11}^{d}$, ${V}_{22}^{d}$ and ${V}_{12}^{d}$ (${V}_{21}^{d}$) are 0.010 eV, 0.020 eV and 0.009 eV, respectively. The spectroscopic constants of diatoms calculated on the new diabatic PESs are consistent with the experimental results. Based on the present diabatic PESs, the TDWP calculations for the Na(3p) + H_2_(X^1^Σ_g_^+^) → NaH(X^1^Σ^+^) + H(^2^S) reaction are carried out to obtain the rigorous quantum dynamics information. The dynamics results show the reaction threshold is consistent with the endothermicity obtained from the diabatic PESs. There exist some oscillation structures on the reaction probability curves due to the complex forming in the potential well. The total ICS steeply rises when the collision energy below 1.5 eV, and the rovibrational states ICSs of the product NaH molecule are presened. In addition, the product NaH molecule tends to be forward scattered, and the forward bias becomes more obvious with increase of the collision energy. As we know, there is no available experimental study which can directly examine the present results. We anticipate our studies could serve as a reference of future experiments for the title reaction.

## Method

### Potential Energy Surface

#### Diabatization method

Several diabatization methods^35–42^ have been developed, and the most important step is to obtain the transformation matrix between the diabatic and adiabatic representations. An effective transformation approach with less calculation burden is to use appropriate molecular properties associated with the transition of electronic states to construct the matrix, and the dipole moment is selected for the NaH_2_ system. A brief description about the diabatization scheme is presented below. In this work, the diabatic coupling includes two electronic states, thus the unitary transformation from the adiabatic basis ${\psi }_{i}^{a}$ to the diabatic basis ${\varphi }_{i}^{d}$ can be expressed as

1 $$(\begin{array}{c}{\varphi }_{1}^{d}\\ {\varphi }_{2}^{d}\end{array})=(\begin{array}{cc}\cos \,\alpha  & -\,\sin \,\alpha \\ \sin \,\alpha  & \cos \,\alpha \end{array})(\begin{array}{c}{\psi }_{1}^{a}\\ {\psi }_{2}^{a}\end{array}),$$

where *α* is called the mixing angle. The energy matrix elements at the diabatic representation is calculated as follows

2 $${V}_{11}^{d}={E}_{1}^{a}\,{\cos }^{2}\,\alpha +{E}_{2}^{a}\,{\sin }^{2}\,\alpha ,$$

3 $${V}_{22}^{d}={E}_{1}^{a}\,{\sin }^{2}\,\alpha +{E}_{2}^{a}\,{\cos }^{2}\,\alpha ,$$

4 $${V}_{12}^{d}={V}_{21}^{d}=({E}_{2}^{a}-{E}_{1}^{a})\cos \,\alpha \,\sin \,\alpha ,$$

where ${E}_{1}^{a}$ and ${E}_{2}^{a}$ are the adiabatic energies of the ground state and first excited state. ${V}_{11}^{d}$ and ${V}_{22}^{d}$ represent the diagonal terms of the diabatic energy matrix, and the values of non-diagonal terms ${V}_{12}^{d}$ and ${V}_{21}^{d}$ are equal for all configurations. From equation 1, the $\langle {\psi }_{3}^{a}|\hat{P}|{\psi }_{1}^{a}\rangle$ and $\langle {\psi }_{3}^{a}|\hat{P}|{\psi }_{2}^{a}\rangle$ can be written as

5 $$\langle {\psi }_{3}^{a}|\hat{P}|{\psi }_{1}^{a}\rangle =\langle {\psi }_{3}^{a}|\hat{P}|{\varphi }_{1}^{d}\,\rangle \cos \,\alpha +\langle {\psi }_{3}^{a}|\hat{P}|{\varphi }_{2}^{d}\,\rangle \sin \,\alpha ,$$

6 $$\langle {\psi }_{3}^{a}|\hat{P}|{\psi }_{2}^{a}\rangle =-\,\,\langle {\psi }_{3}^{a}|\hat{P}|{\varphi }_{1}^{d}\,\rangle \sin \,\alpha +\langle {\psi }_{3}^{a}|\hat{P}|{\varphi }_{2}^{d}\,\rangle \cos \,\alpha ,$$

where ${\psi }_{3}^{a}$ is the adiabatic state 1^2^A″ which is not involved in the electronic coupling, and $\hat{P}$ denotes the dipole moment operator. The assumption is to make $\langle {\psi }_{3}^{a}|\hat{P}|{\varphi }_{1}^{d}\rangle =0$ and $\langle {\psi }_{3}^{a}|\hat{P}|{\varphi }_{2}^{d}\rangle =1$ not just for the collinearity. Thus, the *α* is calculated by

7 $$\alpha =\arctan \,[|\frac{{\langle \psi }_{3}^{a}|\hat{P}|{\psi }_{1}^{a}\rangle }{{\langle \psi }_{3}^{a}|\hat{P}|{\psi }_{2}^{a}\rangle }|].$$

#### *Ab initio* calculations

The electronic structures of the 1^2^A′ and 2^2^A′ adiabatic states for the NaH_2_ system are calculated in *C*_s_ symmetry using the icMRCI + Q method with a complete active space self-consistent field (CASSCF) reference wave function. In the state-averaged CASSCF calculations, three electronic states (1 A′, 2 A′ and 1 A″) of NaH_2_ are assigned to equal weight. Three valence electrons are in fifteen active orbitals (11a′ + 4a′′), consisting of two 1 s orbitals on H, and 3 s, 3p, 3d, 4 s and 4p orbitals on Na. The frozen-core approach is used in the MRCI calculations, and the orbitals (4a′ + 1a′′) are set to close, thus three electrons are included in the calculations of correlation energy. The aug-cc-PVQZ and cc-pWCVQZ basis sets are used for H atom and Na atom, respectively. A total of 32402 geometries are selected to structure the diabatic energy matrix in the Jacobi coordinates. The energy points are defined by 0.6 ≤ *r*_HH_/*a*_0_ ≤ 28, 0 ≤ *R*_Na–HH_/*a*_0_ *≤* 35, 0 ≤ *θ*/degree ≤ 90 for the Na−HH region, and 0.8 ≤ *r*_NaH_/*a*_0_ ≤ 28, 0 ≤ *R*_H–NaH_/*a*_0_ ≤ 35, 0 ≤ *θ*/degree ≤ 180 for the H−NaH region. All of the *ab initio* calculations are implemented by MOLPRO package^43^.

#### Fitting of the diabatic PESs

The energies in the diabatic representation are constructed by combining the adiabatic data with the diabatic transformation based on molecular property method. All of the diabatic energies are used to fit the diabatic terms of ${V}_{11}^{d}$, ${V}_{22}^{d}\,\,$and ${V}_{12}^{d}({V}_{21}^{d})$ by the NN method. The NN method is an excellent tool to accurately establish PES, and has been used to numerous reactive systems^44–50^. The feed-forward NN is employed in this work. The NN consists of a set of input signal {*x*_*i*_}, one output signal corresponded to energy and two hidden layers. The output signal of a neuron can be presented as

8 $${y}_{i}=f(\sum _{j=1}^{N}{w}_{i}{x}_{i}+{b}_{j}),$$

where *w*_*i*_ and *b*_*j*_ are the weights and bias of interconnecting neurons, respectively. The linear function as the transfer function *f(x)* in the output layer, and the hyperbolic tangent function is chosen in the two hidden layers, which is written as

9 $$f(x)=\frac{{e}^{x}-{e}^{-x}}{{e}^{x}+{e}^{-x}}.$$

The permutation invariant polynomials^51,52^ are used in the fitting of each diabatic term. To prevent overfitting, all of the diabatic energies are randomly divided into the training (90%), validation (5%) and testing (5%) sets. The final fitting energy can be expressed as

10 $${V}_{fit\_norm}={f}^{(3)}(\sum _{i=1}^{{N}_{2}}{w}_{i}^{(3)}{f}^{(2)}(\sum _{j=1}^{{N}_{1}}{w}_{j}^{(2)}{f}^{(1)}(\sum _{k=1}^{K={\rm{3}}}{w}_{k}^{(1)}{I}_{k}+{b}_{j}^{(1)})+{b}_{i}^{(2)})+{b}^{(3)}),$$

where the superscripts of (1) and (2) represent the first and second hidden layers, and superscript of (3) represents the output layer. *I*_*k*_ are input data after corresponding geometries preprocessing. For the fitting of ${V}_{11}^{d}$ and ${V}_{22}^{d}$, 13 and 14 neurons are used in each hidden layer, and 10 neurons are used in the fitting process of ${V}_{12}^{d}$ (${V}_{21}^{d}$). The (RMSEs) for ${V}_{11}^{d}$, ${V}_{22}^{d}$ and ${V}_{12}^{d}$ (${V}_{21}^{d}$) are 0.0100 eV, 0.0204 eV and 0.009 eV, respectively.

To examine the accuracy of fitted diabatic PESs, the comparison between spectroscopic constants of H_2_$({{\rm{X}}}^{1}{{\rm{\Sigma }}}_{\,g}^{+})$ and NaH $({{\rm{X}}}^{1}{{\rm{\Sigma }}}_{\,}^{+})$ calculated on the diabatic PESs and experiment data^53–55^ are shown in Table 1. It is obvious that the calculated results are good agreement with the experimental values.

**Table 1:** Spectroscopic constants of H_2_(X^1^Σ_g_^+^) and NaH(X^1^Σ^+^).

		This work	Experiment
H_2_(X^1^Σ_g_^+^)	*R*_*e*_ (bohr)	1.400	1.401
	*D*_*e*_ (eV)	4.723	4.747
	*ω*_*e*_ (cm^−1^)	4403.8	4401.2
	*ω*_*e*_*x*_*e*_ (cm^−1^)	112.2	121.3
NaH(X^1^Σ^+^)	*R*_*e*_ (bohr)	3.647	3.566
	*D*_*e*_ (eV)	1.920	1.971
	*ω*_*e*_ (cm^−1^)	1188.1	1171.9
	*ω*_*e*_*x*_*e*_ (cm^−1^)	18.9	19.7

### Quantum Dynamics

The quantum dynamics simulation for the Na(3p) + H_2_(X^1^Σ_g_^+^) → NaH(X^1^Σ^+^) + H(^2^S) reaction is carried out by the TDWP method on the new NN diabatic PESs, which has been described in detail previously^56,57^. This method is effective to treat the diabatic transition between two electronic states, and only an online involved the main equations is presented below. In the body fixed representation, the Hamiltonian of the Na + H_2_ reaction is written as follows

11 $$\hat{H}=-\frac{{\hslash }^{2}}{2{\mu }_{R}}\frac{{\partial }^{2}}{\partial {R}^{2}}-\frac{{\hslash }^{2}}{2{\mu }_{r}}\frac{{\partial }^{2}}{\partial {r}^{2}}+\frac{{(\hat{J}-\hat{j})}^{2}}{2{\mu }_{R}{R}^{2}}+\frac{{\hat{j}}^{2}}{2{\mu }_{r}{r}^{2}}+\hat{V},$$

where *R* is the distance between Na and HH center of mass, and *r* represents HH bond length. *µ*_*R*_ and *µ*_*r*_ denote the reduced masses relevant to *R* and *r*. *J* and *j* are the total and H_2_ molecular angular momentums. $\hat{V}$ is the diabatic PESs of the NaH_2_ system. The reactant coordinate based method is used to obtain the S-matrix at the product channel, which is developed by Sun *et al*.^58^. The states resolved reaction probability can be calculated by

12 $${P}_{\upsilon j\leftarrow {\upsilon }_{0}{j}_{0}}^{J}=\frac{1}{2{j}_{0}+1}{\sum _{K,{K}_{0}}|{{\rm{S}}}_{\nu jK\leftarrow {\nu }_{0}{j}_{0}{K}_{0}}^{J}|}^{2}.$$

The states selected ICSs and DCSs are calculated by the following equations

13 $${\sigma }_{\upsilon j\leftarrow {\upsilon }_{0}{j}_{0}}=\frac{\pi }{(2{j}_{0}+1){k}_{{\upsilon }_{0}{j}_{0}}^{2}}\sum _{K}\sum _{{K}_{0}}{\sum _{J}({\rm{2}}J+{\rm{1}})|{{\rm{S}}}_{\nu jK\leftarrow {\nu }_{0}{j}_{0}{K}_{0}}^{J\epsilon }|}^{2},$$

14 $$\frac{d{\sigma }_{\upsilon j\leftarrow {\upsilon }_{0}{j}_{0}}(\theta ,E)}{d{\rm{\Omega }}}=\frac{1}{(2{j}_{0}+1)}{\sum _{K}\sum _{{K}_{0}}|\frac{1}{2i{k}_{{\upsilon }_{0}{j}_{0}}}\sum _{J}(2J+1){d}_{K{K}_{0}}^{J}(\theta ){{\rm{S}}}_{\nu jK\leftarrow {\nu }_{0}{j}_{0}{K}_{0}}^{J\epsilon }|}^{2},$$

where *θ* denotes the scattering angle between the incoming Na (3p) + H_2_ and the scattered NaH + H, and ${d}_{K{K}_{0}}^{J}(\theta )$ represents the Wigner rotation matrix.

In this work, only the ground rovibrational state reaction of Na(3p) + H_2_ (*v*_0_ = 0, *j*_0_ = 0) → NaH + H are performed the TDWP calculations, and the main dynamics parameters are given in Table 2 by numerous convergence tests.

**Table 2:** Numerical parameters used in quantum wave packet calculations.

Na(3p) + H_2_ → NaH + H	
Grid/basis range and size	*R* (bohr) ∈ [0.1, 15.0], *N*_*R*_ = 149
	*r* (bohr) ∈ [0.1, 15.0], *N*_*r*_ = 149,
	*N*_*j*_ = 100
Initial wave packet $\exp [-\frac{{(R-{R}_{c})}^{2}}{2{{\rm{\Delta }}}_{R}^{2}}]\cos \,{k}_{0}R$	R_c_ = 10.0 bohr Δ_R_ = 0.14 bohr *k*_0_ = (2*E*_0_*μ*_*R*_)^1/2^ with *E*_0_ = 1.30 eV
Total propagation time	20000 iterations
Time step	15 a.u.
Highest J value	70
